# Total Thickness Bilateral Rupture of the Rectus Abdominis: A Case Report and Literature Review

**DOI:** 10.1155/2024/8868853

**Published:** 2024-07-09

**Authors:** Dernas Suhail, Olivia Smith, Philip Lim, Srinivas Chintapatla

**Affiliations:** ^1^ Hull York Medical School University of York, University Rd, York, UK; ^2^ York Abdominal Wall Unit York Teaching Hospital, Wigginton Road, York, UK

## Abstract

Rupture of the rectus abdominis is a rare condition. We describe the case of a young male trainee paratrooper who presented with sudden onset severe lower abdominal pain that occurred during military training. Magnetic resonance imaging revealed full-thickness bilateral rupture of the rectus abdominis. Our management involved injecting botulinum toxin into the rectus muscles preoperatively, reconstructing the rectus muscles, and placing a supportive biosynthetic mesh. Postoperatively, our patient could walk pain-free at 3 weeks, jog pain-free at 10 weeks, and run up to 2 miles at 25 weeks. As there is no consensus on the management of this rare injury, we conducted a literature review on all cases reporting rupture of the rectus abdominis from the year 2000. After comparing the outcomes of conservatively versus surgically managed patients, we can conclude that generally, management of such defects is dependent on size, severity, and patient factors; however, surgical treatment yields comparable results to conservative treatment.

## 1. Introduction

Muscle injuries are among the most common exercise-related injuries, contributing from 10% to 55% of exercise-related injuries [1]. Hence muscle rupture is neither new nor unusual. But the rare form of muscle rupture seen in our patient makes our case distinctive. Our patient suffered a full-thickness, bilateral rupture of the rectus abdominis, which is a very rare presentation. Previously reported cases of rectus abdominis rupture in the literature have been due to handball, tennis, and other exercise-related injuries, as well as blunt trauma to the abdomen [2, 3, 4, 5, 6, 7, 8, 9]. However, there is no consensus on how to manage this rare injury, and whether surgical or conservative treatment yields better results.

## 2. Case Presentation

Our patient has provided informed consent to publish their case. A male trainee paratrooper in his early 20s presented to our accident and emergency department following an injury sustained during military training. While jumping from a kneeling to a standing position, he heard a distinct tearing sound from his lower abdomen, quickly followed by severe, nonradiating pain in the same area.

To facilitate physical examination, our patient required 10 mg of oramorph. Upon physical examination, while in a supine position, we noted a slight hollowing in his lower abdomen, which was very tender upon palpation. Passive hip flexion was pain-free, but active hip flexion caused severe pain. Furthermore, our patient was not able to sit back up from a supine position. There was no femoral or inguinal hernia, and scrotal examination showed no abnormalities.

**Figure 1:**
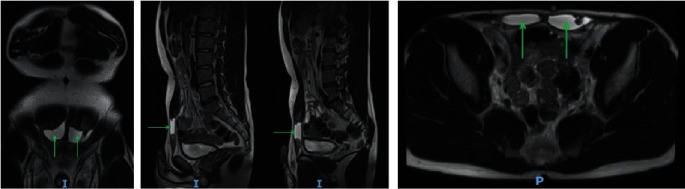
Coronal, sagittal, and transverse magnetic resonance images of the patient, arrows highlight the bilateral defect in the rectus muscles. I, inferior and P, posterior.

Magnetic resonance imaging showed well-defined defects in the distal aspect of both right and left rectus muscles representing full-thickness tears with minor retraction of the proximal muscle (Figure 1). Both defects measured approximately 3 cm in length and contained blood. The distal tendinous insertions into the pubic body, the rectus sheath, and the linea alba remained intact.

Having considered the magnetic resonance imaging (MRI) results, we felt that there was a low chance the defect would heal adequately without surgical intervention. Not only would inadequate healing have implications on activities of daily living, but considering the patient's paratrooper career goals, we deemed surgical intervention to be necessary, in order to facilitate a steady and sufficient rehabilitation back to his army duties.

Botulinum toxin was injected into the patient's rectus muscles under ultrasound guidance 2 days preoperatively to produce a temporary flaccid paralysis. Three hundred units of Dysport-A botulinum toxin were injected on each side, 100 units into the cranial, 100 units into the middle, and 100 units into the caudal bellies of the rectus muscle bilaterally. Using a Pfannenstiel incision, we dissected down to and incised the anterior rectus sheath. Intraoperatively, we noted a partial thickness tear superior to the full-thickness tear on the right rectus muscle. Following this, all nonviable muscle and fascia were debrided. The right rectus muscle was then dissected from the posterior rectus sheath to allow muscle advancement and approximation. Phasix ST mesh, a delayed absorbable biosynthetic mesh, was placed in the retrorectus plane and sutured in place using 3/0 PDS sutures. The objective of this mesh is to provide tensile strength, in order to reinforce the closure. The anterior rectus sheath was closed using 3/0 PDS, and pain-buster pumps were placed around the rectus sheath to provide a continuous, regulated infusion of bupivacaine. The abdominal wound was closed using 3/0 monocryl sutures in Scarpa's fascia and deep dermis. Total negative pressure dressings were placed in situ, set at 88 mHg. The immediate postoperative course was uneventful, and our patient was discharged one week later.

Our patient made good progress in the following weeks. Postoperatively he was able to walk pain-free at 3 weeks. At 10 weeks, he was able to jog and carry out activities of daily living pain-free, such as driving and shopping. At 25 weeks, he could jog up to 2 miles. Also, at this point in time, the scar had healed well, and he could strain the abdominal muscles by lifting both legs simultaneously while in a supine position. At 1-year follow-up, our patient has returned to full army duties and is able to undertake all physical requirements. Sensation around the scar is approximately 7/10 on examination, which is decreased, but sufficient to be protective of injury.

## 3. Discussion

Full-thickness rupture of the rectus abdominis is a rare injury. There is currently no consensus on whether surgical or conservative management yields better outcomes for this condition. Hence, we undertook a literature review with the aim to collate the available literature on the management of this rare presentation. Pubmed, Medline, and Embase databases were searched to identify relevant studies using the following MeSH terms, “rectus abdominis” and “rupture,” as well as their free text counterparts and synonyms. We identified eight other cases of rectus abdominis rupture from the year 2000 [2, 3, 4, 5, 6, 7, 8, 9].

Six articles discussed management using conservative means [2, 3, 4, 5, 6, 7] (Table 1), while two articles chose surgical management [8, 9] (Table 2). Interestingly, all other exercise-related cases in the literature were managed conservatively, which differs from our choice of surgical management for our case with trauma-related aetiology. Conversely, all trauma-related cases in the literature were managed surgically. Among both conservatively and surgically treated patients, none suffered any complications, and all patients returned to normal levels of activity.

Our case is most comparable to the case described by Gozubuyk et al. [2]. Their patient was 25-year-old male who suffered a full-thickness bilateral rupture of the rectus abdominis while flexing his rectus muscles using an abdominal crunch machine at the gym. Thus, the patient demographics, injury aetiology, as well as specific injury features between our patient and Gozubuyuk's patient are very similar. However, their patient was managed conservatively, making for a very compelling comparison of outcomes. The course of recovery was very similar for the two patients. Our patient reported pain-free walking at 3 weeks and pain-free jogging at 10 weeks postoperatively, whereas Gozubuyuk's patient-reported pain-free walking at 4 weeks and pain-free free jogging at 10 weeks into rehabilitation. Both our patient and Gozubuyuk's patient reported a return to activities of daily living pain-free in the 10th week.

Comparing the similar successful outcomes between our patient and this similarly presenting, conservatively managed patient, it is unclear as to whether surgical management yields a better repair for patients with a ruptured rectus abdominis. However, surgical intervention allowed us to identify an additional defect in the muscle not identified through imaging. A similar example found in the literature was of a 12-year-old boy who presented with abdominal pain following blunt abdominal trauma from a bicycle handlebar [9]. Their MRI scan only showed a 1.8 cm defect, but due to omental fatty tissue being palpated on the skin, he underwent immediate surgery, during which his defect was found to be 8 cm in length, much larger than shown on MRI. With MRI not showing the full extent of injury in both our patient's case, as well as the case of this 12-year-old boy, this imaging modality may not be infallible in evaluating such defects.

We deemed it beneficial to preoperatively inject the rectus muscles with botulinum toxin, which is not commonly described in the literature. Botox produces a temporary flaccid paralysis of the abdominal musculature, which allows additional tissue mobility needed to achieve primary closure. Botox also prevents strong contraction of the rectus muscles during the healing process, which could otherwise jeopardize the repair. Compared to other abdominal wall expansion options, botox is associated with the highest primary closure rate, lowest recurrence rate, and lowest complication rate [10, 11].

The patient's career goals in the army were important to keep in mind while devising our management plan, as he would need to gain full preinjury function to achieve such goals. It is known that if the ends of muscle and tendon are left with significant gaping, some remodeling and scarring occur, however functionally, these patients yield suboptimal functional outcomes. In addition, if the gap between the stumps of ruptured muscle is very wide, the denervated muscle may sustain a permanent neurological deficit resulting in muscle atrophy. However, surgical intervention increases the chance of reinnervation and maintenance of optimal function [12]. From our experience, we advise that a surgical approach utilizing the placement of mesh in the retrorectus space is a practical way to repair the abdominal wall in a young patient with a physically demanding career to optimize functional outcomes. Although our findings depict comparable outcomes between conservative and surgical approaches in the short-term, long-term results are needed to make more definitive conclusions.

## 4. Conclusion

Full-thickness bilateral rupture of the rectus abdominis is a rarely seen condition. Our method of preoperatively injecting botulinum toxin into the rectus muscles, followed by reconstruction of the ruptured rectus abdominis and placement of supporting mesh yielded good outcomes, allowing our patient to return to preinjury levels of activity 1 year following reconstruction. Generally, management of such defects is dependent on size, severity, and patient factors; however, conservative treatment yields comparable results to surgical treatment.

## Figures and Tables

**Table 1:** Reported cases of conservatively managed rectus abdominis rupture.

Reference	Patient	Presentation	Imaging findings	Outcomes
Gozubuyuk et al. [2]	25-year-old male	Severe stabbing lower abdominal pain following use of abdominal crunch machine at the gym	MRI—full-thickness bilateral tear of rectus muscles with retracted fibers above and below injury. Distal tendinous insertion into pubis intact	Pain-free walking at the fourth week and pain-free jogging at 10th week of rehabilitation. Returned to daily activities pain-free at 10th week as well
Ruff et al. [5]	14-year-old female	Stabbing right lower quadrant pain following jump box exercises	Ultrasound—partial rupture of right rectus abdominis 1.7 × 3.7 cm. No herniation of bowel content	Able to return to exercise 5 weeks after her injury, with no pain. Follow-up ultrasound at 3 months showed good muscle healing with no complications
Cottaar et al. [3]	24-year-old female	Experienced acute abdominal pain during routine abdominal workout	Ultrasound—bilateral partial rupture of rectus abdominis, 1 cm wide and 0.6 cm deep. Fascia and linea alba intact. No intramuscular haematoma	Follow-ups at 2, 6, and 11 weeks showed good recovery. Patient returned to exercise after 10 weeks with no problems
Minardi et al. [6]	23-year-old female, professional gymnast	3 days history of worsening right lower quadrant pain	Ultrasound—rupture of the right rectus abdominis	No complications and was able to return to her job as a professional gymnast
Tubez et al. [7]	22-year-old male, professional tennis player	Lower abdominal pain felt while the trunk was in extension, entering flexion while swinging a tennis racket	MRI—12 mm tear in distal left rectus abdominis	Patient experienced a new tear on distal insertion of psoas muscle, causing temporary cessation of competition
Balius et al. [4]	All professional handball players (1) 28-year-old male (2) 16-year-old male (3) 27-year-old male (4) 28-year-old male (5) 21-year-old male	NA	Ultrasound showed: (1) Unilateral rupture, 26 × 12 mm (2) Unilateral rupture, 35 × 20 mm (3) Unilateral rupture, 35 × 20 mm (4) Unilateral rupture, 16 × 10 mm (5) Unilateral rupture, 17 × 19 mm	Returned to competitive handball in: (1) 17 days (2) 20 days (3) 16 days (4) 16 days (5) 22 days

**Table 2:** Reported cases of surgically managed rectus abdominis rupture.

Reference	Patient	Presentation	Imaging findings	Outcomes
Patel et al. [8]	45-year-old female, obese	Patient was a front seat passenger in a high velocity road traffic accident and suffered blunt abdominal trauma from impact of the seatbelt	CT—ruptured rectus abdominis, small intestine mesentery damage, small intestine infarction, and large intestine infarction	No complications with regard to repair of the abdominal wall musculature and was able to return to her job as a teacher
Narci et al. [9]	12-year-old male	Patient was subject to blunt abdominal trauma from handlebar impact due to bicycle riding accident	CT—1 × 1.8 cm rupture of right rectus abdominis. Omental fatty tissue was palpated under skin and, hence, was operated on immediately. In surgery, rupture was found to be 8 cm long	Uneventful postoperative course. At 1-month follow-up, there was no denervation, herniation, or rectus diastasis

## Data Availability

Data supporting this case report are not available.
